# Fear of COVID-19 and related factors affecting mothers’ breastfeeding self-efficacy during the pandemic

**DOI:** 10.1590/1980-220X-REEUSP-2022-0130en

**Published:** 2022-10-24

**Authors:** Sibel Ergün, Serap Kaynak, Beray Aydın

**Affiliations:** 1 Balıkesir University, Department of Pediatric Nursing, Faculty of Health Sciences, Balıkesir, Turkey.; 2 Institute of Health Sciences, Balıkesir, Turkey.

**Keywords:** Breast Feeding, Pandemic, Mothers, COVID-19, Self Efficacy, Lactancia Materna, Pandemia, Madres, COVID-19, Autoeficacia

## Abstract

**Objective:**

This study aimed to determine the breastfeeding self-efficacy levels of mothers during the pandemic period, to compare them according to various characteristics, and to examine the relationship between the fear of COVID-19 and breastfeeding self-efficacy.

**Method:**

The data of this descriptive and cross-sectional were collected through a web-based survey of 392 mothers between June and August 2021. The data collection tools were the Introductory Data Form, the Breast-feeding Self-Efficacy Scale-Short Form, and the Fear of COVID-19 Scale.

**Results:**

The mean score for breastfeeding self-efficacy was found to be 56.18 ± 8.24, while the mean score for the Fear of COVID-19 scale was 21.77 ± 6.14. Having a high fear of COVID-19, breastfeeding more frequently in this period, and suspecting that they had COVID-19 affected the breastfeeding self-efficacy scores positively, whereas graduating from primary school had a negative effect on self-efficacy.

**Conclusion:**

The breastfeeding self-efficacy of mothers who were fearful of COVID-19, who breastfed more frequently during the pandemic, and who had a higher education level were positively affected.

## RESUMEN

**Objetivo:**

Este estudio tuvo como objetivo determinar los niveles de autoeficacia de lactancia materna durante la pandemia, compararlos según diversas características y examinar la relación entre el miedo al COVID-19 y la autoeficacia de lactancia materna.

**Método:**

Los datos del presente estudio descriptivo y transversal se recopilaron a través de una encuesta basada en la web con 392 madres, entre junio y agosto de 2021. Las herramientas de recopilación de datos fueron el Formulario de Datos de Introducción, el Formulario Breve de Escala de Autoeficacia de Lactancia Materna y la Escala de Miedo al COVID-19.

**Resultado:**

La puntuación media de autoeficacia de lactancia materna fue de 56,18 ± 8,24, mientras que la puntuación media de escala de miedo al COVID-19 fue de 21,77 ± 6,14. Tener un gran miedo al COVID-19, amamantar con mayor frecuencia en este período, y sospechar de COVID-19 afectó positivamente los puntajes de autoeficacia de lactancia materna, mientras que graduarse de la escuela primaria tuvo un efecto negativo en la misma autoeficacia.

**Conclusión:**

Conclusión: Se afectó positivamente la autoeficacia de lactancia materna de aquellas que tenían miedo al COVID-19, que amamantaron con mayor frecuencia durante la pandemia y que tenían un mayor nivel educativo.

## INTRODUCTION

Breast milk reduces neonatal, infant, and child mortality. As this milk contains all the nutrients and energy needed for growth, it is a food that is easy to digest and has strong biological benefits. At the same time, it does not require preparation, is always available at the appropriate temperature, is economical and reliable, and protects the baby against infections^(1)^. The World Health Organization (WHO) has declared that “breastfeeding is very important for the survival, nutrition and healthy development of infants”. Along with other organizations the WHO has stated that infants should be exclusively breastfeeding for the first six months^(2)^.

According to the research, most women start to feed their babies with breast milk alone immediately after birth, but this rate decreases as the baby grows. Only 36% of 0-6-month-old babies in the world are fed with breast milk. The WHO has reported that the lives of more than 820,000 children under the age of 5 could be saved each year if children are breastfed until they are 2 years old^(3)^. According to the 2018 data of the Population and Health Survey in Turkey, 59% of 0-1-month-old children, 45% of 2-3-month-old children, and 14% of 4-5-month-old children are exclusively breastfed^(4)^.

The aim of global nutrition policies is to increase exclusive breastfeeding by 50% or more for the first six months of life worldwide by 2025^(5)^. Knowing the factors affecting breastfeeding is important to achieve this goal. According to the literature, factors such as social support, age, education level, and being married affect breastfeeding^(6)^. One of the most important factors in continuing to breastfeed is that breastfeeding mothers have a perception of breastfeeding self-efficacy^(7)^. Self-efficacy describes a mother’s perceived ability and self-confidence to breastfeed her baby. This sense of self-efficacy affects breastfeeding in the first six months after birth. A high perception of breastfeeding self-efficacy is very effective in maintaining breastfeeding. A feeling of inadequacy and lack of self-confidence are among the most important negative factors affecting the effectiveness of breastfeeding^(6)^.

The coronavirus, which emerged in China, spread across many countries within three months, despite the implementation of intensive isolation and quarantine measures. The WHO declared a worldwide pandemic in March^(8)^. The coronavirus (COVID-19) can affect individuals from birth onwards. Given the statements of the Academy for Breastfeeding Medicine (ABM) and the recommendations of the WHO, the general trend during coronavirus infections is to continue to breastfeed^(1)^.

During the COVID-19 pandemic, restrictions and social distancing changed breastfeeding behaviors for mothers^(9)^. When mothers receive support for successful breastfeeding, it is effective in many areas, including ensuring skin-to-skin contact after birth, keeping the mothers and baby together, and starting breastfeeding immediately. Therefore, receiving and maintaining breastfeeding support is very important for successful breastfeeding^(10)^. However, it was observed that breastfeeding support decreased during the pandemic period. The WHO suggested that the mother and baby stay in the same room and have skin contact, and that the mother continue breastfeeding while wearing a surgical mask and paying attention to hand hygiene. Since the numerous benefits of skin-to-skin contact and breastfeeding for the baby are often greater than the risk of COVID-19 in children, which are often asymptomatic or mild, WHO made various recommendations for breastfeeding mothers regarding COVID-19^(11)^. However, at the beginning of the pandemic, it was unknown whether coronavirus would be transmitted from mother to child prenatal, postpartum, or through breastfeeding^(12)^. In addition, posts on social media claiming that breastfeeding was not safe caused concern in society at large. During the planning phase of this research, no study was found in Turkey investigating mothers’ attitudes towards breastfeeding and their choices and how they were affected by the process. It is important to analyze the breastfeeding self-efficacy of mothers during the coronavirus pandemic, and provide evidence for strategies and nursing practices to be developed to meet their needs. In addition, it has been stated in the literature that SARS-CoV-2 antibodies, which protect against COVID-19, provide passive immunity in infants and that breastfeeding is safe during the pandemic. Breastfeeding during the pandemic should thus be a priority for both mothers and babies^(13)^. Importantly, there is IgA and IgG in the milk of sick mothers. These substances prevent the formation of SARS-CoV-2. Therefore, it has been recommended that mothers with COVID-19 continue breastfeeding^(14)^. For these reasons, our study was designed to identify some predictors of breastfeeding self-efficacy during this period.

## METHOD

### Type of Study

This study was descriptive, correlational, and cross-sectional.

### Purpose of Study

This study aimed to (1) determine the breastfeeding self-efficacy levels of mothers during the pandemic period, (2) compare them with the fear of COVID-19 and breastfeeding behaviors, and (3) reveal their relationship with breastfeeding self-efficacy. Within this scope, answers were sought to the following research questions:

Q1: Are mothers’ fear of COVID-19 related to breastfeeding self-efficacy?

Q2: Are the breastfeeding behaviors of mothers related to breastfeeding self-efficacy?

Q3: Are mothers’ education levels and being diagnosed with COVID-19 during pregnancy related to breastfeeding self-efficacy?

**Figure 1:**
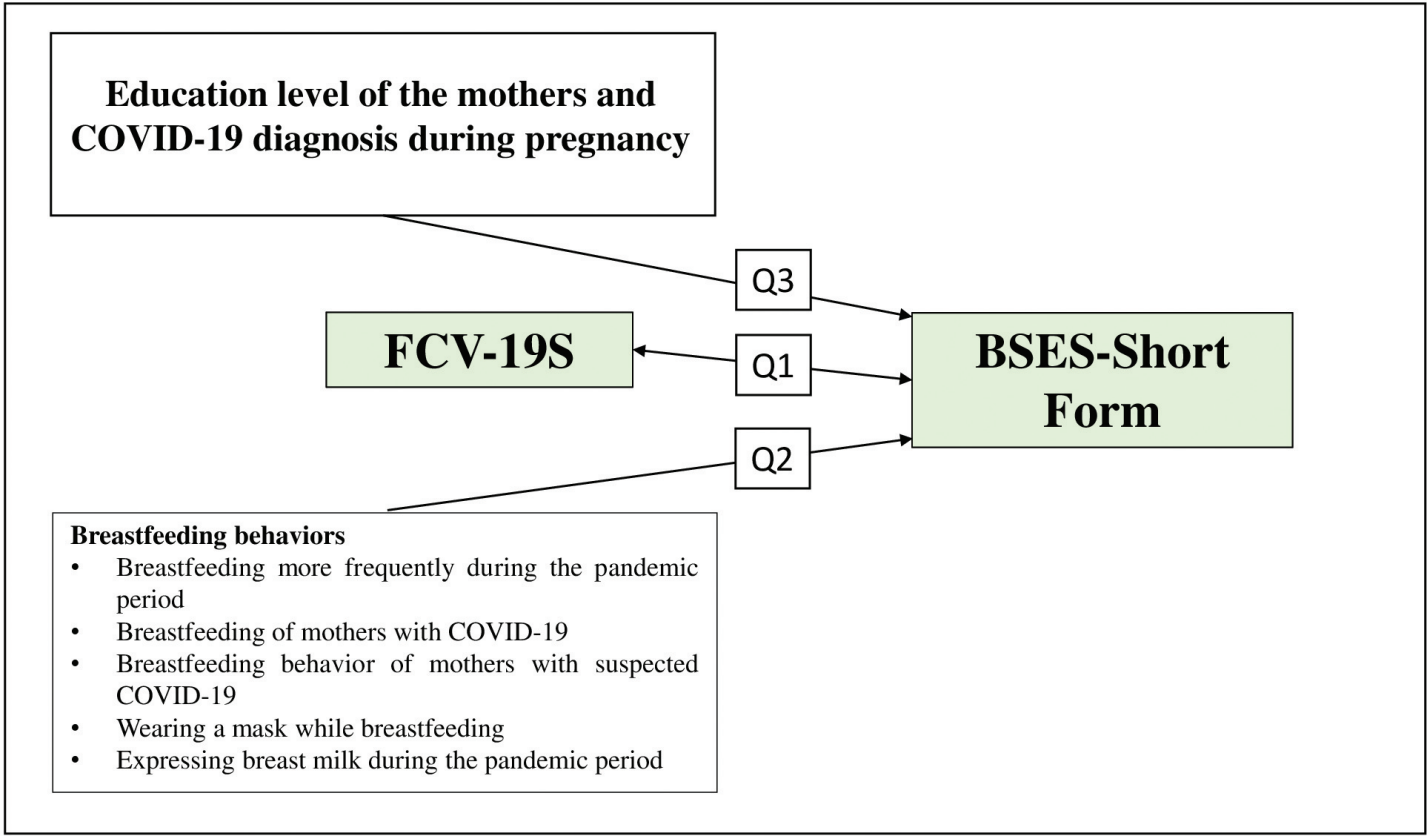
Research model.

In line with the research questions, the research model is given in Figure 1.

### Population

In calculating the sample, the formula used to determine the number of individuals to be sampled was used to examine the incidence of the event. Since the number of people in the population was not known in the study, the formula n = (t2 X (Pq) / d2) was used and the number of participants was calculated as 384^(15)^. In the study, 392 people who agreed to the take part in the study filled out online questionnaires. The study sample thus consisted of 392 people. A 0.95% confidence interval, 5% standard deviation, and 50% unknown prevalence were used for calculations.

### Inclusion Criteria

The selection criteria for the mothers were that they were women with no mental or physical illness and no chronic disease, were over 18 years old, had a baby aged 0–24 months, were breastfeeding the baby and were literate.

### Data Collection

The data were collected from 5 June to 6 August 2021. Data were collected using an online Google Form. The universe of the research consisted of mothers over 18 years old who used social media (Facebook, WhatsApp), and agreed to participate in the research. There was an informed consent form on the first page of the questionnaire. Those who agreed to participate were able to continue to the other pages with the data collection tools after they had read the form and confirmed that they were participating voluntarily. The mothers who filled out the questionnaire were asked to share it with other breastfeeding mothers. Accordingly, an accessible link to the online data collection created in Google Docs was provided via social networks.

## INSTRUMENTS

### Personal Data Form

The researchers prepared the form in line with the literature^(6, 9, 16, 17, 18)^. The content of the form included questions about the mother’s age, the baby’s age, education, profession, residence, economic situation, breastfeeding by mothers with COVID-19, breastfeeding behaviors of mothers with suspected COVID-19, wearing a mask while breastfeeding, and diagnosis of COVID-19 during pregnancy.

### Breastfeeding Self-Efficacy Scale-Short form (BSES-SF)

This scale was created to measure how competent mothers feel about breastfeeding. It is a 14-item scale. On a five-point Likert scale, 1 represents the minimum, and 5 the maximum score. The points for each item are added together and the total score is obtained. The scale score ranges between 14 and 70. As the score obtained from the scale increases, self-efficacy also increases^(17, 18)^. The scores obtained from the scale are grouped as demonstrating low (14–32), medium (33–51), and high (52–70) self-efficacy^(16, 19)^.

### Fear of COVID-19 Scale (FCV-19)

This scale was created to measure the fear caused by COVID-19. It is a seven-item one-dimensional scale. On a five-point Likert scale, 1 represents the minimum and 5 the maximum score. The points for each item are added together and the total score is obtained. The scale score ranges between 7 and 35. As the score obtained from the scale increases, fear of COVID-19 also increases^(20, 21)^.

### Ethical Aspects

Ethics approval was obtained from the Ethics Committee (Date: 14.04.2021, No: 2021/113). The participants were informed about the study and it was stated that filling in the form voluntary. The mothers clicked on the “I approve” button on the screen featuring the online questionnaire.

### Data Analysis

The data of the study were transferred to the computer environment and analyzed using the IBM SPSS Statistics 21 package program. Number, percentage, and mean were used in the analysis of basic descriptive data. Multiple regression, in which all predictors were entered simultaneously, was used to examine factors associated with mothers’ breastfeeding self-efficacy. Before analyzing the data, assumptions of normality, covariance, linearity, and multicollinearity between independent variables were tested. During the normality analysis, the values of the skewness and kurtosis variables were evaluated. For the multicollinearity test of the analyses, correlation values were analyzed to ensure that there was no multicollinearity between the variables. Since all values of variables were found to be less than 0.7, which is an acceptable range for a correlation coefficient, no predictor was considered to be multicollinear. Tolerance and VIF were used to control for possible multicollinearity between predictive variables. The tolerance values for the variables in the equation range were between .591 and .960, while VIF values ranged from 1.042 to 1.738.

## RESULTS

### Prevalence and Demographic Characteristics

Table shows that 56.4% of the participants were university graduates and 63.3% were living in an urban area. In addition, 53.8% of mothers were housewives and 84.7% were living in a nuclear family. 20.4% of the mothers had COVID-19 during breastfeeding and 76.5% thought that breast milk protected the baby.

### Mean Score for Breastfeeding Self-Efficacy and Fear of COVID-19

The total mean score for breastfeeding self-efficacy was found to be 56.18 ± 8.24, while the mean score for fear of COVID-19 was 21.77 ± 6.14 (see Table 1).

**Table 1:** Mean Scores for Breastfeeding Self-Efficacy and Fear of COVID-19 – Balıkesir, Marmara Region, Turkey, 2021.

	**Items**	**Min–Max**	**X**	**SD**
**BSES–SF**	14	14–70	56.18	8.24
**FCV–19S**	7	7–35	21.77	6.14

Abbreviations: Max: maximum; Min: minimum; SD: standard deviation; X: Mean.

### Factors Related to Breastfeeding Self-Efficacy and Fear of COVID-19

The age of mothers ranged from 18 to 45 and the mean age was 29.53 ± 4.99. The babies were between 1–24 months old and the mean age was 9.56 ± 5.73 (months). Although the mean scores of the mothers who graduated from university were higher than those who graduated from high school and primary school, statistical significance was found only with breastfeeding self-efficacy. The scores of the mothers living urban areas for both scales were higher than the mothers living in villages and towns. However, statistical significance was found only with the fear of COVID-19 (see Table 2).

**Table 2:** Characteristics of mothers and comparison of breastfeeding self-efficacy and fear of COVID-19 – Balıkesir, Marmara Region, Turkey, 2021.

**Variables**	**Frequency**	**Breastfeeding self-efficacy, mean (SD)**	**Fear of COVID-19, mean (SD)**
**Mother’s age (y)** (avg. age: 29.53 ± 4.99 (min:18, max:45))			
**Baby’s age (m)** (avg. age: 9.56 ± 5.73 (min:1, max:24))			
**Education**			
Primary school	50 (12.8)	52.96 (8.75)	21.84 (4.87)
High school	221 (56.4)	55.68 (8.74)	21.36 (6.54)
University	121 (30.9)	58.43 (6.33)	22.50 (5.84)
		F = 9.113; p = .000,	F = 1.355; p = .259
		1 < 3^*^, 2 < 3^*^	
**Occupation**			
Housewife	211	56.10 (8.17)	6.00 (0.41)
Government employee	135	56.02 (8.01)	6.33 (0.54)
Self-employed	46	57.04 (9.30)	6.23 (0.91)
		F = .285; p = .752	F = .960; p = .384
**Residence**			
Village	19 (4.8)	54.05 (9.96)	19.15 (7.00)
District	125 (31.9)	55.50 (8.59)	21.15 (3.37)
Urban area	248 (63.3)	56.69 (7.90)	22.29 (5.90)
		F = 1.537; p = .216	F = 3.272; p = .039,
			3 < 1*, 3 < 2*
**Economic Status**			
Low-income	29	55.44 (6.77)	23.65 (6.00)
Middle-income	342	56.26 (8.17)	21.64 (6.06)
High-income	21	55.95 (11.15)	21.23 (7.43)
		F = .139; p = .870	F = 1.512; p = .222

Note: ^*^F: Test of one-way ANOVA.

Abbreviations: ANOVA: analysis of variance; SD: standard deviation, p < .05, p < .01.

### Bivariate Correlations Between Breastfeeding Self-Efficacy and Maternal Breastfeeding Behaviors In COVID-19 Pandemic

The means and standard deviations of the variables and their intercorrelation values are given in Table 3. There was a significant correlation between breastfeeding self-efficacy (r = .148, p = .003) and the fear of COVID-19 during the pandemic period. Additionally, a significant correlation was determined between breastfeeding self-efficacy (r = .197, p = .000) and the breastfeeding behavior of mothers with suspected COVID-19 during the pandemic period.

**Table 3:** Bivariate correlations between breastfeeding self-efficacy and maternal breastfeeding behaviors in the COVID-19 pandemic – Balıkesir, Marmara Region, Turkey, 2021.

	**M (SD)**	**1**	**2**	**3**	**4**	**5**	**6**	**7**
1. Breastfeeding self-efficacy	56.18 (8.24)	1						
2. Fear of COVID-19	21.77 (6.14)	.148^**^	1					
3. Having COVID-19 while breastfeeding	0.204 (0.40)	–.013	.039	1				
4. Expressing breast milk during the pandemic	0.096 (0.29)	–.091	.022	.155^**^	1			
5. Breastfeeding behavior of mothers with suspected COVID-19	0.760 (0.42)	.197^**^	.094	.136^**^	.043	1		
6. Mother with COVID-19 breastfeeding her baby while masked	0.257 (0.43)	–.041	.191^**^	.150^**^	.182^**^	–.011	1	
7. Breast milk protects baby from COVID-19	0.765 (0.42)	.048	.109^*^	.041	.059	.154^**^	.106^*^	1

Pearson correlation analysis.

### Predictive Factors of Participants' Breastfeeding Self-Efficacy

Error term analysis obtained from the stepwise multiple regression model created with the BSES-SF total score independent variable showed that the data provided normality, linearity, and homoscedasticity assumptions (Durbin Watson: 1.75). Educational status and independent variables affecting breastfeeding behaviors during the pandemic were included in the multiple regression, which was used to determine the variables affecting breastfeeding self-efficacy, and a model was created. Accordingly, four independent variables were determined to be significant in the model. These were education level, COVID-19 fear level, included in breastfeeding behaviors; breastfeeding more frequently during the pandemic period, and suspecting that one had COVID-19. This model explained 17 % of the variance in breastfeeding self-efficacy. In the study, we found that a high level of COVID-19 fear, breastfeeding more frequently during the pandemic, and breastfeeding by those with suspected COVID-19 affected breastfeeding self-efficacy positively, while graduating from primary school affected the breastfeeding self-efficacy negatively (see Table 4).

**Table 4:** Multiple regression result on BSES-SF score – Balıkesir, Marmara Region, Turkey, 2021.

**Variables**	**Unstandardized Standardized**			**t**	**p**	**95% CI**
	**B**	**SE**	**β**			
(Constant)	41.500	2.135		19.441	.000	37.30 to 45.69
Fear of COVID-19	.178	.063	.133	2.811	.005^**^	.05 to .30
Breastfeeding more frequently during the pandemic (1 = Yes)	11.442	1.615	.333	7.085	.000^***^	8.26 to .14.61
Breastfeeding by mothers with COVID-19 (1 = Yes)	–1.890	1.369	–.084	–1.381	.168	–4.58 to 0.80
Breastfeeding behavior of mothers with suspected COVID-19 (1 = Yes)	3.208	1.154	.166	2.779	.006^**^	.93 to 5.47
Wearing a mask while breastfeeding (1 = Yes)	–.957	.900	–.051	–1.063	.288	–2.72 to 0.81
Education level (1:Primary school)	–3.068	1.159	–.124	–2.646	.008^**^	–5.34 to –.78
Expressing breast milk during the pandemic (1 = Yes)	–1.307	1.318	–.047	–.992	.322	–3.89 to 1.28
COVID-19 diagnosis during pregnancy (1 = Yes)	–.204	1.309	–.007	–.156	.876	–2.77 to –2.37

Note: R = .435, R^2^ = .190, Adjusted R^2^ = .173, F = 11.194, ^*^p < .05. ^**^p < .01. ^***^ p < .001. df 8.383, Durbin Watson:1.75.

## DISCUSSION

Breastfeeding is an effective health protection practice, the success of which is strongly related to breastfeeding self-efficacy^(22)^. This study aimed to identify predictors of breastfeeding self-efficacy during the period of the pandemic.

The mean scores obtained from the scales in the current study were compared with the literature. Scores obtained from BSES-SF scale are grouped as showing low (14–32), medium (33–51), and high (52–70) self-efficacy^(16, 19)^. In this study, the mean breastfeeding self-efficacy score was 56.18 ± 8.24. Mean scores for breastfeeding self-efficacy vary between 47.10 and 61.12 in the literature^(6, 16, 23)^. This study determined that breastfeeding self-efficacy was high. Antenatal and postnatal care are provided free of charge in Turkey. Therefore, women benefit from these health services. We think that this is the reason for the high level of breastfeeding self-efficacy discovered.

The mean score for the FCV-19S was found to be 21.77 ± 6.14. Mean scores for this scale vary between 18.00 ± 4.30 and 19.44 ± 6.07 in the literature^(19, 24, 25)^. Studies have reported that, due to the pandemic, mothers are worried about both their babies and their own health^(26)^. In one study, it was determined that about a quarter of mothers showed clinical symptoms of anxiety and depression symptoms during the pandemic^(27)^. In our study, the mothers’ high level of fear was similar to that found in the literature.

The breastfeeding self-efficacy of mothers who were university graduates was higher in the current study. In the literature, a statistically significant difference was found between the postnatal period and the education level of the mothers during the pandemic period^(28)^. We can attribute this to the fact that the participants with a high level of education were more aware of the importance of breastfeeding during the pandemic, and more conscientious about breastfeeding their babies.

Although the mothers living in urban areas had higher breastfeeding self-efficacy and fear of COVID-19 than those living in villages and towns, statistical significance was found only with the fear of COVID-19. We can attribute this to the fact that mothers living in urban areas had more information about COVID-19 because it is easier to access information and communication technologies there. In addition, life in the city means encountering more crowds in places such as stations, subways, or shopping malls.

In our research, a positive and significant relationship was found between breastfeeding self-efficacy, the fear of coronavirus and suspecting that one had COVID-19. Results showed that the fear increased breastfeeding self-efficacy in mothers. Fear is the body’s natural response to danger. It can be chronic and severe during periods of pandemic. Patients who suspect they have COVID-19 may show behavioral and emotional reactions (anger, insomnia, loneliness, impatience, fear or anxiety etc.). Some mothers may worry about their relatives, and this can increase their fear. In a study in Turkey examining the psychosocial impact of the pandemic on preschool children and their mothers, it was found that mothers experienced various negative emotions during the pandemic, the most common emotion of which was the fear of losing family members and relatives, which increased their fear and anxiety levels significantly. Additionally, the same study found that there was also frequent anxiety about the future^(29)^. Likewise, studies shown that fear, anxiety, and worry is frequently seen in mothers during the pandemic^(26, 30)^.

In the study, we determined that having a high level of COVID-19 fear, breastfeeding during the pandemic, and suspecting that one had COVID-19 positively affected breastfeeding self-efficacy, while graduating from primary school negatively affected breastfeeding self-efficacy. The effects of the pandemic on breastfeeding have differed from the impact of other crises and epidemics. It can be concluded that mothers continued breastfeeding during the pandemic and followed the recommendations of experts in order to try to strengthen their baby’s immune system, due to the fear of their baby catching COVID-19.

## Limitations of The Study

The fact that the research data was only obtained from mothers living in Balıkesir can be considered as a limitation of the study. In addition, the cross-sectional collection of data while the COVID-19 pandemic was still ongoing can be seen as another limitation.

## CONCLUSION

In conclusion, although the findings of literature are similar to the findings of our study in terms of mean breastfeeding self-efficacy and predictors, it is important that this study was conducted during a period of pandemic. The results of the current study showed that having a high level of COVID-19 fear, breastfeeding more frequently during the pandemic, and suspecting that one had COVID-19 affected breastfeeding behavior positively, whereas graduating from primary school had a negative impact on breastfeeding behavior.

The results of this study once again demonstrate the importance of providing breastfeeding training to mothers during the pandemic, using an interdisciplinary and multidisciplinary approach including teamwork. Nurses can provide training by explaining the importance of breastfeeding to mothers during the pandemic, using visual breastfeeding materials, and making use of simulations. Conducting individual interviews with mothers will allow their specific educational needs to be determined and met. This, in turn, will help strengthen breastfeeding behaviors during the still-ongoing COVID-19 pandemic.

## References

[1] Gökçay G, Keskindemirci G. (2020). Breastmilk and Covid-19. J Ist Fac Med.

[2] Kornides M, Kitsantas P. (2013). Evaluation of breastfeeding promotion, support, and knowledge of
benefits on breastfeeding outcomes. J Child Health Care.

[3] World Health Organization (2016). World breastfeeding week [Internet].

[4] Hacettepe University Institute of Population Studies (2018). Population and Health Survey in Turkey [Internet].

[5] World Health Organization (2014). Global Nutrition Targets 2025: Breastfeeding policy [Internet].

[6] Ahmad Zadeh Beheshti M, Alimoradi Z, Bahrami N, Allen KA, Lissack K. (2022). Predictors of breastfeeding self-efficacy during the covid-19
pandemic. J Neonatal Nurs.

[7] Baud D, Qi X, Nielsen-Saines K, Musso D, Pomar L, Favre G. (2020). Real estimates of mortality following COVID-19
infection. Lancet Infect Dis.

[8] Haykır N. (2020). Breastfeeding and COVID-19 Pandemic. South Clin Ist Euras.

[9] Brown A, Shenker N. (2021). Experiences of breastfeeding during COVID‐19: lessons for future
practical and emotional support. Matern Child Nutr.

[10] Gavine A, McFadden A, MacGillivray S, Renfrew MJ. (2017). Evidence reviews for the ten steps to successful breastfeeding
initiative. J Health Visit.

[11] World Health Organization (2020). Frequently Asked Questıons: Breastfeeding and COVID-19 For health care
workers [Internet].

[12] Juan J, Gil MM, Rong Z, Zhang Y, Yang H, Poon LC. (2020). Effects of coronavirus disease 2019 (COVID‐19) on maternal,
perinatal and neonatal outcomes: a systematic review. Ultrasound Obstet Gynecol.

[13] Bäuerl C, Randazzo W, Sánchez G, Selma-Royo M, García Verdevio E, Martínez L (2022). SARS-CoV-2 RNA and antibody detection in breast milk from a
prospective multicentre study in Spain. Arch Dis Child Fetal Neonatal Ed.

[14] Pace RM, Williams JE, Järvinen KM, Belfort MB, Pace CD, Lackey KA (2020). COVID-19 and human milk: SARS-CoV-2, antibodies, and neutralizing
capacity. MedRxiv [Preprint].

[15] Sümbüloglu K, Sümbüloglu V. (2007). Biostatistics.

[16] Öztürk R, Ergün S, Özyazıcıoğlu N. (2022). Effect of antenatal educational intervention on maternal
breastfeeding self-efficacy and breastfeeding success: a quasiexperimental
study. Rev Esc Enferm USP.

[17] Dennis CL. (2003). The breastfeeding self-efficacy scale: psychometric assessment of
the short form. J Obstet Gynecol Neonatal Nurs.

[18] AluşTokat M, Okumuş H, Dennis CL. (2010). Translation and psychometric assessment of the breast-feeding
self effi cacy scale short form among pregnant and postnatal women in
Turkey. Midwifery.

[19] Dodou HD, Bezerra RA, Chaves AFL, Vasconcelos CTM, Barbosa LP, Oriá MOB. (2021). Telephone intervention to promote maternal breastfeeding
self-efficacy: randomized clinical trial. Rev Esc Enferm USP.

[20] Ahorsu DK, Lin CY, Imani V, Saffari M, Griffiths MD, Pakpour AH. (2022). The Fear of COVID-19 Scale: development and initial
validation. Int J Ment Health Addict.

[21] Bakioğlu F, Korkmaz O, Ercan H. (2021). Fear of COVID-19 and positivity: mediating role of intolerance of
uncertainty, depression, anxiety, and stress. Int J Ment Health Addict.

[22] Saghooni NM, Barez MA, Moeindarbari S, Karimi FZ. (2017). Investigating the breastfeeding self-efficacy and its related
factors in primparous breastfeeding mothers. Int J Pediatr.

[23] Karbandi S, Hosseini SM, Masoudi R, Mamori GA. (2014). The effect of relaxation training on breastfeeding self-efficacy
of mothers with preterm infants: a randomized clinical trial. J Clin Nurs Midwifery.

[24] Altundağ Y. (2021). Fear of Covid-19 and resillience during the early Covid-19
pandemic. Journal of EKEV Academic.

[25] Uzun A, Öztürk GZ, Bozkurt Z, Çavuşoğlu M. (2021). Investigating of fear of COVID-19 after pregnancy and association
with breastfeeding. JIDHealth.

[26] Terada S, Kinjo K, Fukuda Y. (2021). The relationship between postpartum depression and social support
during the COVID-19 pandemic: a cross-sectional study. J Obstet Gynaecol Res.

[27] Fernandes DV, Canavarro MC, Moreira H. (2021). Postpartum during COVID‐19 pandemic: portuguese mothers’ mental
health, mindful parenting, and mother–infant bonding. J Clin Psychol.

[28] Durmuş A, Öztaş HG. (2022). Knowledge levels of breastfeeding and the effect of breastfeeding
self-suffıciency on mothers who have been diagnosed or contacted with
COVID-19. Kirsehir Ahi Evran University Journal of Health Sciences.

[29] Tuzcuoğlu N, Aydın D, Balaban S. (2021). Investigation of the case of pandemic’s psychosocial ımpact on
preschool children based on mothers’ views. J Res Elem Educ.

[30] Matvienko‐Sikar K, Meedya S, Ravaldi C. (2020). Perinatal mental health during the COVID‐19
pandemic. Women Birth.

